# Editorial

**DOI:** 10.1007/s41109-016-0002-3

**Published:** 2016-06-01

**Authors:** Ronaldo Menezes, Hocine Cherifi

**Affiliations:** Florida Institute of Technology, Melbourne, FL USA

Data mining and machine learning have long enhanced our understanding of patterns in data. Yet, data is most often structured in ways that correspond to existing relationships between parts of the data. Understanding the structures formed from data relationships often leads to increased clarity regarding the application areas represented by the data. Network science aims at understanding, modeling, and predicting the dynamics of this structure (a network) and the dynamics in the network (the network as an enabler of information transmission). Moreover, the massive amount of data today requires the development of tools and algorithmic techniques to handle networks of unimaginable sizes. Networks with millions of nodes and links are now the norm rather than the exception. There is currently a shortage of efficient tools and algorithms for analyzing such networks.

The field of network science (aka complex networks) has made huge strides in creating theories that help us to understand both the dynamics *of* networks (its structure) and *in* the network (processes using networks as a framework). The success of the field is such that many applied areas have embraced the power of networks. It is now clear that behind most complex systems, there is a structure (a network) that governs the dynamics of such a system.

Networks are ubiquitous to most parts of society; we live in social networks (we are social animals) and use online social networks as means of communication (social media, phone networks, SMS, etc.); we deal with malicious threats (cyber-attacks to computer networks, terrorism, disruptions to infrastructure, etc.); we use resources such as electricity and the Internet; the structure of these networks relate directly to their resilience; we worry about pandemics and the spread of disease as a function of the connectivity of networks (of contacts between people, flight patterns); networks of authors, musicians, actors, and athletes may help us understand what leads to success in these fields; to name just a few examples. Ultimately, understanding networks may enable better solutions to such problems as well as applications to other complex systems.

The journal *Applied Network Science* is intended to focus on applied research benefiting from or using network science. The breadth of areas where network science is being used continues to increase and is far from reaching its peak. Annual meetings on network science continue to attract a diverse crowd—from physicists to urban planners; from computer scientists to art historians. These works contribute to the body of knowledge of applications which can benefit from network science.

We have set the scope of this journal to be on “applied” work exactly to highlight the multi- and inter-disciplinary aspects of the journal. We encourage contributions from diverse fields as long as the contributions are not solely theoretical. Papers should clearly indicate how the concepts proposed can be applied to practical, real-world problems. Note that we are open to papers with theoretical results, but there should be a clear indication in the body of the work about the applied impact of the proposed theory.

Our first submissions are currently being reviewed and we expect a quick turn-around. Many other submissions are being prepared. We invite you to submit your work to demonstrate the world-wide applicability of network science.

Hocine Cherifi and Ronaldo Menezes

Editors-in-Chief

